# Do not compromise: Nurse honeybees practice strict protein-lipid regulation

**DOI:** 10.1016/j.isci.2025.112895

**Published:** 2025-06-17

**Authors:** Pierre Lau, Pierre Lesne, Alexandria N. Payne, Cora Garcia, Jordan Gomez, Spencer T. Behmer, Juliana Rangel

**Affiliations:** 1 Department of Entomology, Texas A&M University, College Station, TX 77845, USA; 2 United States Department of Agriculture, Pollinator Health in Southern Crop Ecosystem Research Unit, Stoneville, MS 38776, USA

**Keywords:** Entomology, Exotic species behavior, Animal nutrition

## Abstract

The Nutritional Geometry Framework (NGF) has been instrumental in revealing how animals regulate nutrient intake. In honeybees (*Apis mellifera*), most research has emphasized protein-carbohydrate regulation, even though pollen is rich in both protein and lipid. We used NGF-based no-choice and choice experiments to examine how nurse bees, responsible for brood care, regulate protein and lipid intake. Bees consumed the most and developed the largest hypopharyngeal glands on diets with 30% protein and 20% lipid. When given dietary options, they regulated intake toward this 1.5:1 protein-lipid ratio. Feeding typically stopped once either a protein or lipid threshold was reached, consistent with a “strict restraint” rule that may reflect physiological costs of even slight overconsumption. These findings underscore the importance of protein-lipid regulation in nurse bees. This work broadens understanding of nutrient regulation in animals that consume protein- and lipid-rich foods and highlights the importance of diet quality for bee health.

## Highlights

• The Nutritional Geometry Framework advances our understanding of animal nutrient regulation
• Poor nutrition in honeybees (*Apis mellifera*) is not properly defined
• NGF no-choice and choice experiments show that nurse bees regulate their protein-lipid intake
• Nurses obey a “strict restraint” rule, ceasing to feed once protein/lipid thresholds are hit

## Graphical abstract

**Figure:**
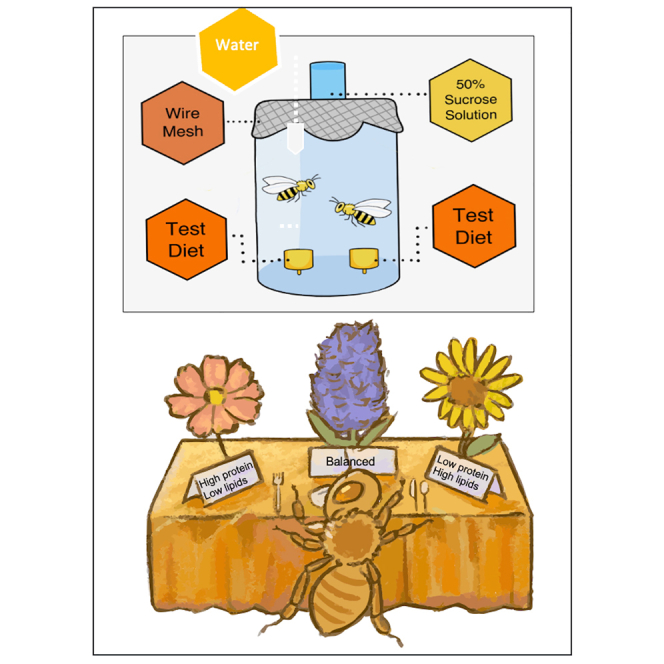

## Introduction

Nutrition is a fundamental driver of animal health, development, and ecological success, influencing everything from metabolic function to behavioral strategies.^1^^,^^2^^,^^3^ Central to these processes is effective nutrient regulation, which ensures homeostasis, optimizes fitness, and enables adaptation to environmental challenges.^4^^,^^5^ In general, all animals require a broad suite of ∼30 nutrients.^1^^,^^2^ When the intake of key macro- and micronutrients is balanced, growth, survival, longevity, immunity, and reproduction are functionally optimal.^1^^,^^2^^,^^3^^,^^4^^,^^5^ Our current understanding of animal nutrient regulation has been greatly informed by the Nutritional Geometry Framework (NGF), a powerful modeling approach based on the representation of food consumption in a multidimensional nutritional space.^6^^,^^7^ The NGF has mostly been used to explore the effects of protein and carbohydrate intake on survival, growth, reproduction, and overall health, identify species-specific self-selected protein and carbohydrate intake targets (IT; the optimal combination and amount of protein and carbohydrates that an animal needs to consume to meet its physiological needs), and reveal the regulatory strategies (i.e., rules of compromise) adopted by species when preferred protein-carbohydrate intake targets cannot be reached.^4^^,^^6^^,^^7^ The NGF has also been extended to other nutrients and used to explore nutrient-toxin interactions.^4^^,^^5^^,^^6^^,^^7^ Although the NGF was initially applied to study nutrient regulation at the individual level, it has been successfully used to study nutrient foraging strategies in animals that have complex social systems,^8^ and those that forage collectively, including ants^9^^,^^10^^,^^11^^,^^12^ and bees.^13^^,^^14^^,^^15^^,^^16^

For animals that forage collectively, especially eusocial insects, nutrient regulation occurs at multiple levels.^17^^,^^18^^,^^19^ First, active foragers – typically older adults – make up only a small subset of the total number of individuals in the collective group. While foragers feed themselves, they are also tasked with collecting food for all the non-forager nestmates. Second, the non-foragers, which comprise both immatures and adults, have different nutritional demands: while immatures require nutrients that fuel growth, adults generally require nutrients linked to energy and maintenance because they are no longer actively growing. Third, the nutritional demands of adults shift as they mature, depending on the age-based division of labor (i.e., age polyethism) of a particular species.^20^ Honeybees (*Apis mellifera*) are an example of this form of nutrient regulation, as younger adult bees (i.e., nurses), who consume a diet of mostly pollen that is rich in protein and lipids, shift to a diet of mostly honey and nectar that is rich in carbohydrates when they become older (i.e., foragers) as a result of age polyethism.^20^^,^^21^

For honeybees, poor nutrition is often linked to the reported yearly losses of managed colonies.^22^^,^^23^ However, what constitutes poor nutrition for honeybees is still inadequately defined. The health of a honeybee colony is best reflected in its brood, whose well-being directly depends on the nutritional health of the nurse bees that tend and feed them.^24^^,^^25^ Nurse bees, in addition to consuming nectar for energy, consume “bee bread” (the stored version of pollen) as the main protein and lipid source. Nurse bees ingest bee bread to develop their hypopharyngeal glands (HPG), which produce the jelly that is fed directly to the developing larvae.^26^ The type and amount of protein eaten by nurses is important for HPG development and is correlated with successful brood rearing.^27^^,^^28^ Lipids, the other major components of pollen, are likely also important for HPG development and brood rearing success. However, little is known about how lipids, and fatty acids in particular, are correlated with either (but see Minahan et al.^29^). Given how pollen is stored within the brood nest, and that it often represents a blend of pollen types, nurse honeybees have the opportunity to selectively forage on it, potentially targeting specific nutrient compositions. This selective foraging may allow them to regulate their intake of key nutrients, especially protein and fatty acids, to support optimal physiological function and brood care.

Historically, there has been an emphasis on protein intake because it has been shown to limit honeybee growth.^18^^,^^30^^,^^31^ However, a singular focus on protein is problematic given the multidimensional nature of nutrition. More recently, a handful of studies have investigated how variation in protein and carbohydrate amounts and ratios (e.g., Paoli et al.^15^; Bouchebti et al.^16^; Hendriksma and Shafir^32^) and protein-lipid ratios (e.g., Stabler et al.^33^; Arien et al.^34^^,^^35^) affect honeybee feeding behavior and performance. To date, the majority of the NGF work in honeybees has focused on protein and carbohydrate regulation. However, there is increasing interest in lipids, given that pollen is rich in both protein and lipids, and lipids are being recognized as nutritionally important for bees.^35^^,^^36^^,^^37^ For example, Stabler et al.^33^ used liquid diets to study honeybee nurse protein-lipid regulation, with a focus on essential amino acids (EAA) as a protein substitute. Nurses showed tight regulation of EAA intake, but very loose regulation of lipid intake. In a more recent study, Minahan et al.^29^ tested the hypothesis that the consumption of an unbalanced diet with a high omega-6:omega-3 ratio would affect nurse bee behavior and the timing of transitioning into a nursing role. They found that feeding nurses a diet with a suboptimal omega-6:omega-3 ratio delayed the onset of nursing and altered their visitation patterns and feeding behavior toward developing larvae. Nutrient regulation has also been investigated in bumble bees. One study found that bumble bees consistently preferred nutrient-rich pollen,^38^ while another showed that a low-nutrient pollen diet led to reduced brood mass and diminished constitutive immunity.^39^ Interestingly, bumble bees consistently select pollen from plant species with specific protein-lipid ratios,^40^^,^^41^^,^^42^ suggesting an innate preference for protein-lipid intake.

Our understanding of honeybee nutrient regulation is in its infancy, and many important questions still remain. Given the nutritional importance of both proteins and lipids, and their abundance in pollen, we hypothesized that nurse honeybees would: (1) actively regulate their protein and lipid intake in no-choice experiments, and (2) selectively eat from two nutritionally complementary foods to obtain an optimal P:L ratio (intake target) while maximizing their protein-lipid intake. Using an NGF approach, we conducted two experiments with nurse honeybees using artificial diets that closely resembled bee bread. For our no-choice experiment, we used five artificial diets that varied in their protein-lipid ratio and then characterized food consumption patterns, protein-lipid regulation strategies, hypopharyngeal gland (HPG) size, body lipid content, and survival of caged nurse bees. For our choice experiment, we used the same diets to identify the protein-lipid intake target and measured how tightly nurse bees regulated it. Our findings offer new insights into how nurse bees regulate their protein-lipid intake and underscores the crucial role of dietary lipids in honeybee nutrition. Additionally, they contribute to broader nutritional frameworks,^43^^,^^44^ especially for animals such as palynivores, predators, and omnivores that commonly consume protein- and lipid-rich foods.

## Results

### No-choice NGF experiment

#### Total food and macronutrient consumption

**Figure 1:**
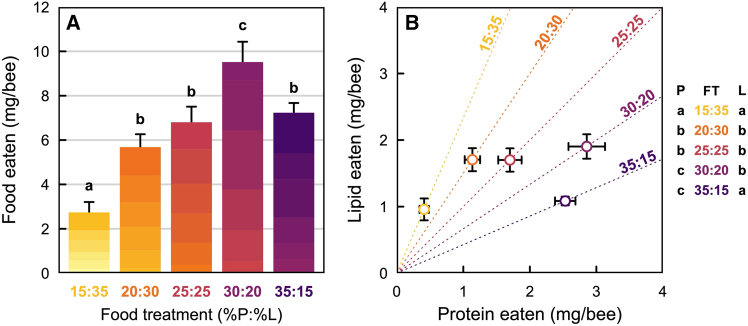
Food consumption by honeybee nurses in laboratory conditions when constrained to a single available artificial diet (no-choice experiment) (A) Average mass of food eaten per bee (mg/bee) over a six-day experiment as a function of the food treatment (mean ± SEM). Food treatments (FT) are noted according to their protein (P) and lipid (L) content (in % values). Each bar is divided into six sections of increasing color intensity representing the daily amount of food eaten per bee on each of the six days of the experiment. Foods represented in yellow are more lipid rich; foods represented in purple are more protein rich; those in between yellow and purple lie within the two extreme values. Different letters above each bar represent significant differences between the per-capita amounts of food eaten over the six days. (B) total protein-lipid (P:L) intake at the conclusion of the six-day experiment under each of the five diet treatments (mean ± SEM). Dotted lines represent the P:L ratio of each artificial diet. Statistical differences for each artificial diet are given on the right of the figure under (P) for the protein axis, and under (L) for the lipid axis. Different letters represent significant differences.

The protein-lipid content of the artificial diets had a significant effect on the amount of food consumed by nurse bees (ANOVA: F_4,55_ = 23.02, *p* < 0.001; Figure 1A). Over the six days of experiment, nurse bees showed the highest consumption when they were fed the P30:L20 diet and had the lowest consumption when they were fed the P15:L35 diet (our most lipid-rich diet treatment). The other diet treatments (P20:L30, P25:L25 and P35:L15) were consumed in intermediate quantities.

Analysis of the total amounts of protein and lipid consumed per bee showed significant differences between treatments (ANOVA for protein: F_4,47_ = 32.92, *p* < 0.001; ANOVA for lipid: F_4,47_ = 7.08, *p* < 0.001; Figure 1B). Protein intake was equally high for the two most protein-rich diets (P30:L20 and P35:L15), and lowest on the most protein-poor diet (P15:L35). Interestingly, lipid intake was equally highest for the three intermediate diets (P20:L30, P25:L25, and P30:L20). In contrast, lipid intake was equally lowest on the two most extreme diets (P15:L35 and P35:L15).

#### Survival, HPG size, and total body lipid

**Figure 2:**
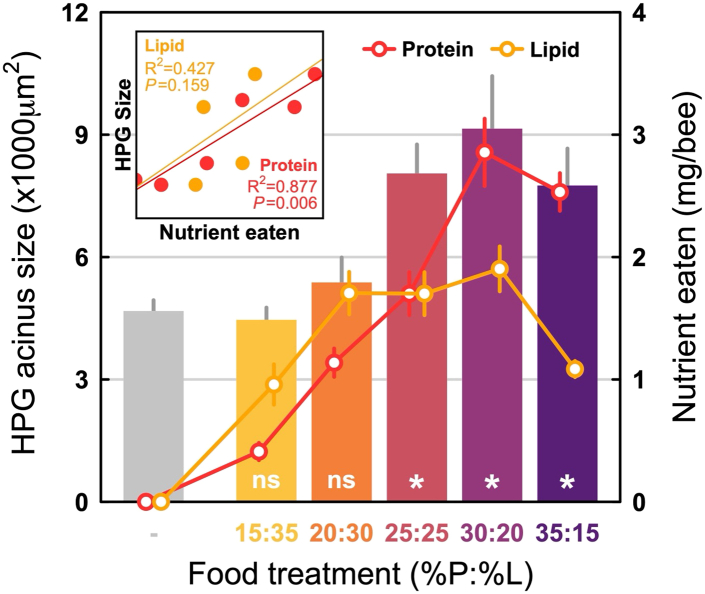
Average hypopharyngeal gland (HPG) acinus size of honeybee nurses (left y axis; bars represent the mean ± SEM HPG acinus size) in the no-choice experiment as a function of the artificial diets (colored bars) compared to the control group (gray bar; bees fed sucrose syrup only) Diets represented in yellow were more lipid rich; diets represented in purple were more protein rich. Lines represent the per capita amount of protein (red line) and lipid (orange line) eaten per bee (mg/bee) as a function of each diet (right axis, Mean ± SEM). The white characters at the bottom of the bars represent whether differences between a given diet and the control group were significant (∗) or not significant (ns). The inlay box shows the relationship between average HPG acinus size and the amount of protein (red) and lipid (orange) eaten per bee and provides the R^2^ and *p*-values for the linear regressions.

We found no effect of a diet’s P:L content on bee survival (Cox mixed-effects model: d.f. = 4, χ^2^ = 2.84, *p* = 0.585). On average, ∼15% of the bees in each cage died over the six-day course of the experiment for all diet groups. Diet P:L content did significantly influence the average HPG acinus size (ANOVA: F_5,66_ = 8.02, *p* < 0.001; Figure 2). Bees that were fed the three most protein-rich diets (P25:L25, P30:L20 and P35:L15) had significantly larger acini than those in the no-food control treatment. Average HPG acinus size of bees from the two most protein-poor treatments (P15:L35 and P20:L30) did not differ compared to the no-food control group. HPG acinus size was positively correlated with protein intake (R^2^ = 0.877, *p* = 0.006) but not with lipid intake (R^2^ = 0.427, *p* = 0.159; Figure 2). Despite differences in lipid intake across the five diets (see above), we found no significant differences in the total lipid content of individual bees, including those from the no-food treatment (ANOVA: F_5,71_ = 1.80, *p* = 0.13; Figure S1).

### Choice NGF experiment

#### Macronutrient consumption

**Figure 3:**
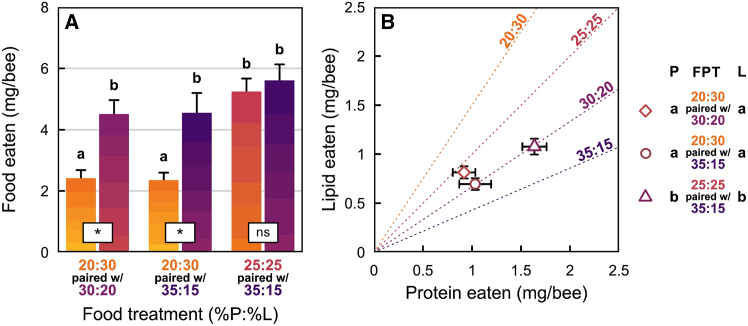
Food consumption by honeybee nurses in laboratory conditions when offered a choice between two artificial diets (choice experiment) (A) Average mass of food eaten per bee (mean ± SEM) over a six-day experiment as a function of the food-pairing treatments (FPT) containing different protein-lipid (P:L) ratios: FPT1 = P20:L30 paired with P30:L20; FPT2 = P20:L30 paired with P35:L15; and FPT3 = P25:L25 paired with P35:L15. Food-pairing treatments are noted according to their protein and lipid content (in %). Each bar is divided in six sections of increasing intensity representing the daily amount of food eaten per capita on each of the six-day experiment. Diets represented in yellow are more lipid rich; diets represented in purple are more protein rich. Different letters above each bar represent significant differences between the per-capita amounts of food eaten over the six days. (B) Total protein-lipid (P:L) intake (mean ± SEM) at the conclusion of the six-day experiment under each of the three diet pairings (circle: P20:L30 paired with P35:L15; triangle: P25:L25 paired with P35:L15; and diamond: P20:L30 paired with P30:L20). The dotted lines represent the P:L ratio of each diet. Statistical differences for each food-pairing treatment (FPT) are given on the right of the figure under (P) for the protein axis, and under (L) for the lipid axis. Different letters represent significant differences.

The bees’ overall diet consumption differed significantly both between (Mixed ANOVA: F_2,33_ = 9.22, *p* = 0.001) and within (F_1,33_ = 30.34, *p* < 0.001) food-pairing treatments. There was also a significant interaction effect between the proteins and lipids consumed (F_1,33_ = 4.47, *p* = 0.019). Total consumption was significantly greater on the protein biased food-pairing treatment (FPT 3: P25:L25 with P35:L15) compared to the two other food-pairing treatments (FPT 1: P20:L30 with P30:L20 and FPT 2: P20:L30 with P35:L15; pairwise *t*-tests; Figure 3A); there was no difference in total consumption between FPT 1 and FPT 2 (pairwise *t*-test; Figure 3A). Next, we showed that for both FPT 1 (P20:L30 with P30:L20) and FPT 2 (P20:L30 with P35:L15), the most protein-rich diet was always consumed in larger quantities compared to the lipid-rich alternative diet (*t*-tests: d.f. = 11, *t* = 4.67, *p* = 0.002; d.f. = 11, *t* = 4.68, *p* = 0.002, respectively). In contrast, for FPT 3 (P25:L25 with P35:L15), the amounts eaten from the two dishes were similar (d.f. = 11, *t* = 0.79, *p* = 1.0).

Next, we looked at diet consumption in individual dishes. For the P25:L25, P30:L20, and P35:L15 diets, consumption was similar across the three food-pairing treatments (pairwise *t*-tests; Figure 3A); in contrast, the P20:L30 diet was consumed in significantly smaller amounts (pairwise *t*-tests; Figure 3A). Interestingly, the P20:L30 diet appeared in multiple food pairing treatments (FPTs), but their amounts eaten were not impacted by their food pairing (P30:L20 or P35:L15).

We detected significant differences in the combined amounts of protein and lipid consumed across our three food-pairing treatments (MANOVA multivariate response: F_2,66_ = 14.89, *p* < 0.001; Figure 3B). The univariate responses showed that these differences were significant along the protein axis (ANOVA univariate response: F_2,33_ = 11.58, *p* < 0.001) and lipid axis (ANOVA univariate response: F_2,33_ = 6.74, *p* = 0.004). These differences were explained by an overall significantly higher consumption of both protein and lipid on FPT 3 (P25:L25 with P35:L15) compared to FPT 1 and FPT 2 (Figure 3B). The self-selected P:L ratios also differed significantly between food-pairing treatments (ANOVA: F_2,33_ = 40.80, *p* < 0.001). FPT 2 (P20:L30 with P35:L15) and FPT 3 (P25:L25 with P35:L15) showed similar P:L consumption ratios (1.52 ± 0.03 P:L and 1.45 ± 0.04 P:L, respectively), and both were significantly more protein-rich than the P:L consumption ratio (1.13 ± 0.02 P:L; Figure 3B) of FPT 1 (P20:L30 with P30:L20).

**Figure 4:**
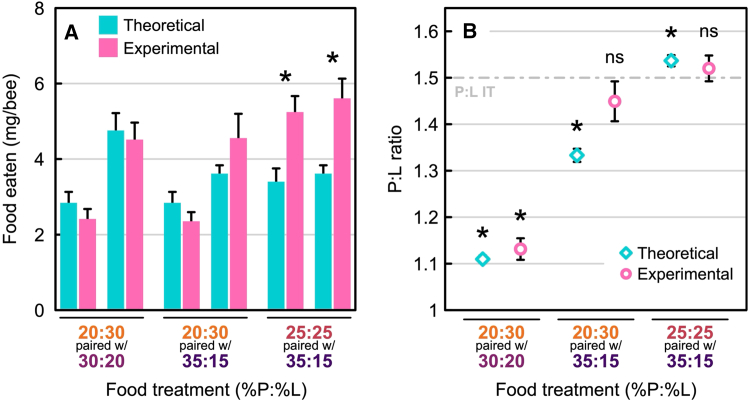
Influence of the food-pairing treatment on the amount of food eaten per bee and the corresponding protein-lipid (P:L) ratio of consumption (A) comparison of the expected food eaten (in mg of food per capita over the six days of the experiment) in absence of intake regulation (turquoise bars: theoretical data) and the food eaten in the choice experiment (pink bars: experimental data) as a function of the food-pairing treatment. The theoretical data were generated based on the amounts of each diet eaten from the no-choice experiment: these values represent the amounts eaten by test bees when the nutritional imbalances of the food cannot be compensated by the consumption of another food. The experimental data are the amount of each food consumed during the choice experiment: these values are the amounts of food eaten by bees when the individuals can self-select their diet from the two available foods. All values are expressed as means ± SEM. A significant difference between the theoretical and experimental data is represented by an asterisk above the pair of bars (with α = 0.0083% after Bonferroni correction of the *p*-values); this supports the idea that food collection is influenced by the nature of the food-pairing and provides evidence for protein-lipid regulation. (B) comparison of the P:L ratios expected in the absence of intake regulation (turquoise diamonds: theoretical data) and the P:L ratios measured experimentally from the choice experiment (pink circles: experimental data). All values are expressed as means ± SEM. The asterisks above the points represent a significant difference, with the target P:L ratio of 1.5 (with α = 0.0083% after Bonferroni correction of the *p*-values).

Lastly, we took a deeper dive into the extent to which nurse bees actively and tightly regulate their protein-lipid intake. First, we observed that bees on FPT 1 (P20:L30 with P30:L20) and FPT 2 (P20:L30 with P35:L15) consumed the two diets in a non-random fashion (one-sample *t*-tests: d.f. = 11, *t* = 4.68, *p* = 0.002; d.f. = 11, *t* = 4.67, *p* = 0.002, respectively). However, on FPT 3 (P25:L25 with P35:L15), the feeding pattern was not statistically different from random (d.f. = 11, *t* = 0.79, *p* = 1.0). Second, we compared the “theoretical” consumption (i.e., what would be expected in the absence of P:L intake regulation) to the “experimental” consumption (i.e., what would be expected when bees regulate their P:L intake) for each diet, and in each food-pairing treatment (Figure 4). In FPT 3 (P25:L25 with P35:L15), the bees collected significantly larger amounts of each diet compared to the theoretical values (*t*-tests: d.f. = 20.6, *t* = 3.35, *p* = 0.019; d.f. = 14.8, *t* = 3.51, *p* = 0.019; respectively; Figure 4A). In contrast, for FPT 2 (P20:L30 with P35:L15), there was no significant departure from the theoretical prediction (Figure 4A). However, for this food-pairing treatment, there was a trend for the experimental values to be: 1) lower compared to the theoretical values with the P20:L30 food, and 2) higher compared to the theoretical values with the P35:L15 food. This translated to an experimental P:L ratio equal to the target P:L ratio, while the theoretical P:L ratio differed significantly from it (one-sample *t*-tests: d.f. = 131, *t* = −11.88, *p* < 0.001; d.f. = 11, *t* = −1.18, *p* = 0.262 for the theoretical and experimental data, respectively; Figure 4B). Finally, while the theoretical P:L consumption ratio for FPT 3 (P25:L25 with P35:L15) was significantly higher than the target P:L ratio (d.f. = 131, *t* = 3.05, *p* = 0.003; Figure 4B), we did not observe a difference between the experimental P:L ratio and the target P:L ratio (d.f. = 11, *t* = 0.72, *p* = 0.484; Figure 4B).

#### Survival

There was no influence of the food-pairing treatment on bee survival (Cox mixed-effect model; difference with the null model: d.f. = 2, χ^2^ = 0.03, *p* = 0.986).

## Discussion

Nurse honeybees have a very specialized and critically important job within the colony: feed the developing brood. Thus, how they regulate their nutrient intake can have profound consequences, especially at the colony level. Using artificial diets that closely resemble the nutrient content and texture of pollen, we show that the feeding behavior in nurse honeybees is strongly influenced by the protein and lipid content of their diets. In our no-choice experiments, the diet with 30% protein and 20% lipid (P30:L20) was eaten in the greatest amounts (Figure 1A). When consumption was visualized as the simultaneous intake of protein and lipid, nurse bees strictly regulated their protein and lipid intake, and generally ate until they reached a threshold level for either protein or lipid (Figure 1B). Importantly, they regulated lipids more tightly than protein. HPG size was also maximized on the P30:L20 diet and was positively correlated with protein but not lipid consumption (Figure 2); furthermore, in the short-term diet (six days of experiment), protein-lipid content did not impact survival. Finally, in the choice experiment, nurse bees generally regulated their protein-lipid intake to a P1.5:L1 ratio (Figure 3B). However, their total protein-lipid intake was reduced when a lipid-rich food was present as a choice (see treatments with P20:L30 foods, Figure 3A). Below we discuss the significance and implications of these results.

Honeybee foragers collect pollen from flowers and store it in their hive as bee bread; this is in turn what nurse bees consume and transform into brood food. The protein and lipid content of pollen is known to vary greatly as a function of plant species; protein content has been reported between 2% and 60%, while lipid content has been reported between 2% and 20%.^18^ More recently, two systematic investigations of pollen P:L ratios^45^^,^^46^ showed a broad protein-lipid spectrum, ranging from lipid-biased (1:9) to balanced (1:1) to protein biased (as high as 29:1). Additionally, evidence suggests that P:L ratios vary phylogenetically across plant families.^45^^,^^47^ In our experiments, we used artificial diets that always contained 50% protein plus lipid and had P:L ratios that varied from 1:2.3 (P15:L35) to 2.3:1 (P35:L15). A comparison of our treatments with the P:L ratios reported from 82 different plant species across 30 different plant families^45^ indicated that our treatments encompassed 67–74% of the P:L values reported in that study (Document S1). Of the pollen sampled by Vaudo et al.^45^ that fell outside of our treatment range, 23–31% had P:L ratios exceeding 2.3:1 (i.e., that of our P35:L15 treatment). In contrast, only 2.4% had a P:L ratio below 1:2.3 (i.e., that of our P15:L35 treatment). Thus, our range of treatments was highly biologically relevant, encompassing the lower and mid-range of P:L ratios. However, we did not capture the highest P:L range. In our no-choice experiment, nurse bees consumed the P30:L20 diet in the largest amounts. Remarkedly, this P:L ratio matches that of the pollen that honeybees naturally collected in the field.^45^ As the P:L ratios increased or decreased around the P30:L20 diet, consumption dropped off, especially on the most lipid-rich diet (P15:L35).

We can best understand nurse bee food consumption patterns across our different treatments by examining their protein-lipid intake patterns (Figure 1B). When animals are confined to single diets, they must balance the costs of overeating nutrients in excess of demands versus the costs of undereating nutrients that are in deficit. The NGF has identified a number of “rules of compromise”^4^^,^^5^ that animals employ in no-choice situations and these rules often reflect some underlying species-specific biology. In the case of nurse honeybees responding to diets with varying protein-lipid content, various outcomes were possible. First, they could eat until their protein intake was met, regardless of their lipid intake (Figure S2, purple triangles, purple line). Second, they could eat until their lipid requirement was met, regardless of their protein intake (Figure S2, yellow triangles, yellow line). The challenge with either of these two rules is that nurse bees would either undereat or overeat the nutrient that is not being regulated, and for most animals there are costs associated with under- or overeating nutrients.^4^^,^^5^ Third, they could compromise by slightly overeating the nutrient in excess, while undereating the nutrient in deficit. Overeating and undereating errors are smaller when animals practice the “closest distance” rule (Figure S2, magenta diamonds) compared to the “equal distance” rule (Figure S2, orange circles). These two “rules of compromise” are consistently seen in insect herbivores in response to protein-carbohydrate regulation.^4^ Nurse honeybees, however, seemed to employ a previously unobserved nutrient regulation strategy. As seen in Figure 1B, they practiced a “strict restraint” rule, whereby they stopped feeding once they reached a threshold intake level of protein *or* lipid. This suggests that for nurse bees, there is a cost of ingesting too much protein *or* lipid, and this likely explains why consumption of the P30:L20 diet was the greatest. On this diet, nurse bees could maximize both their protein and lipid intake. Again, it is notable that this treatment aligns almost perfectly with the P:L ratio of pollen naturally collected by honeybees.^45^ Two additional notes regarding the range of our diet treatments are worth discussing. First, our P15:L35 treatment was extreme in terms of its lipid content (see Document S1). We suspect that the high lipid content made this food less palatable, driven by a combination of pre- and post-ingestive feedback processes.^48^^,^^49^^,^^50^ Second, we did not have a treatment with an extremely high P:L ratio. Given that protein-biased pollen is the norm, ideally, we would have included a P40:L10 treatment. However, when we designed our study, Vaudo et al.^45^ had not yet been published. Regardless, strong evidence already suggests that honeybee nurses tightly regulate their protein intake. Stabler et al.^33^ demonstrated this conclusively by using essential amino acids as a proxy for protein. Their study suggested that nurse bees exhibit strict protein regulation, especially when lipid levels are low.

For nurse bees, the protein and lipid content of pollen/bee bread is important for glandular development.^26^^,^^27^ The HPG of nurse bees in our no-choice experiment were largest when they fed on the diets that had balanced or protein-biased P:L ratios (Figure 2). In contrast, the HPGs of nurses fed on diets with lipid-biased P:L ratios were smaller but equivalent to the sucrose-only control group. This positive correlation between protein intake and HPG size (Figure 2) is consistent with previous studies.^51^^,^^52^^,^^53^ Interestingly, after six days, we saw no differences in total fat body content in nurse bees despite significant differences in dietary lipid availability. Currently, our understanding of lipid uptake and lipid accumulation in adult honeybees is limited. Most of the ingested lipids from pollen are in the form of tryacylglycerides (TAGs)^54^; they comprise a glycerol backbone plus three fatty acids (FA). Following ingestion, the FAs from TAGs are released in the midgut via lipolysis.^55^ Our results suggest that lipid uptake in the midgut occurs at very low levels.

Pollen also contains a small pool of free FAs (FFAs). In honeybees, the FFAs in the lumen of the midgut can be absorbed into the intestinal wall of adults, with amounts increasing over the first three days of adult life and reaching a maximum at day eight, before decreasing continuously in older foragers.^56^ However, the bulk of lipids in adult honeybees are found stored in fat body trophocytes, located in the abdomen, and much of this is a product of larval nutritional history. Interestingly, Corby-Harris et al.^57^ showed that while the ratio of linoleic and linolenic acid differed in the abdomen of newly eclosed adult and nurse bees, total lipid levels did not. Likewise, Scofield and Amdam^58^ showed that lipid levels in the abdomen of adult honeybees do not change significantly over time (i.e., 3, 8, 13, and 21 days post eclosion). Collectively, these three studies suggest that when lipid accumulation does occur in adult honeybees it is primarily in the midgut tissue. Our results suggest that body lipid content is independent of dietary lipid levels, as the lipid values observed across our five treatments did not differ from those of the sucrose-only control bees. This further implies that adult bees generally do not assimilate dietary lipids, as has been observed in other hymenopterans.^59^ Finally, in the short-term (a six-day experiment), we observed no effect of a diet’s P:L ratio on bee survival. Stabler et al.^33^ showed that nurse bees died at a high rate when P:L ratios were very low (1:25), but our most lipid-biased treatment (1:2.3; P15:L35) never approached this level.

The no-choice experiment showed that nurse bees maximize their protein-lipid intake and generate larger HPGs when eating diets that have a P:L ratio of 1.5:1. Thus, when given an opportunity to self-select their own P:L ratio, nurse bees should: (1) choose a P:L intake target of 1.5:1 and (2) maximize their protein-lipid intake. Nurse bees mostly regulated to a P:L ratio of 1.5:1. As seen in Figure 3B, the P:L intake ratio for two of our food-pairing treatments (FPT 2 and FPT 3) fell on the P30:L20 food rail. However, the P:L intake ratio of FPT 1 fell slightly off this rail and was more lipid-biased. Nurse bees on this treatment could have only reached the P1.5:L1 intake target if they fed exclusively on the P30:L20 diet; clearly, this did not happen. With respect to total protein-lipid intake, nurse bees did not regulate to a single protein-lipid intake point (Figure 3B). Instead, total protein-lipid intake was much higher on FPT 3. Interestingly, on FPT 1 and FPT 2, the consumption of individual diets was similar to those from the no-choice experiment (Figure 4A, turquoise: no-choice data, pink: choice data). In contrast, on FPT 3 (P25:L25 with P35:L15), consumption of the two diets exceeded the amount that was calculated for the no-choice experiment. Collectively, this suggests that the protein-lipid content of the diets available to nurse bees has a significant impact on their protein-lipid regulation behavior. Specifically, total protein-lipid intake increased when diet choices encompassed the P:L intake target, but only in the absence of lipid-biased foods.

The implications of our results are perhaps best understood in light of the protein-lipid profiles of pollen.^45^^,^^46^ Our FPT 3 best reflects this range and, on this treatment, nurse bees selected to a P:L ratio of 1.5:1 and maximized their protein-carbohydrate intake. In contrast, total protein-lipid intake on FPT 1 and FPT 2 —both of which contained the P20:L30 diet— was constrained. For nurse bees, this highlights the importance of foragers. They should (1) collect pollen types that have complementary P:L ratios and (2) ignore pollen that is lipid-biased. Again, based on field collected data, this appears to be the case.^45^ This puts nurse bees in a more favorable protein-lipid landscape. In the hive, they would have the opportunity to eat selectively from bee bread that is comprised of a mixture of pollen types, maximizing both their protein-lipid intake and HPG development (Figure 2). One additional point is worth considering. Nurse bees consumed the P20:L30 diet even when a superior diet option was available. This could reflect the nature of colony living, which includes having to make feeding decisions in the context of competing with multiple nurse bees foraging for bee bread. Each of our arenas contained two food dishes that served 25 nurse bees. Because the protein-biased food dish was more preferred, it likely received more visitors. However, given their small size, each dish could only serve a small number of foraging nurse bees. On the other hand, less of the P20:L30 diet was eaten, so it likely received fewer visitors. Perhaps nurse bees consumed the P20:L30 diet because it was readily assessible. However, this choice came at a cost, as it resulted in a lower total protein-lipid intake. Behavioral observations that track individual nurse bees in choice experiments would offer deeper insights into the dynamics of group foraging. Additionally, they could reveal the extent to which individual nurse bees engage in diet-switching between complementary nutritional resources.

Our no-choice and choice experiments indicate that nurse bee feeding behavior is influenced by both protein and lipid content of their food. However, our data suggest that nurse bees regulate lipid intake more strictly than protein intake. In the no-choice experiment, lipid intake exhibited less variation than protein intake (Figure 1B). This pattern is further supported by a more detailed analysis of protein-lipid regulation, using an approach originally developed to investigate protein-carbohydrate regulatory rules in desert locusts.^60^ Figure S3 illustrates the relationship between nurse bees over- or under-consuming protein or lipid relative to the experimentally inferred protein-lipid intake target (IT) from our no-choice and choice experiments. According to the “strict restraint” rule, neither protein nor lipid are ever overconsumed beyond its respective IT. It is worth highlighting that deviation from the intake target was smaller for lipid intake than for protein intake, with significant differences from the theoretical expectation occurring only on the two most extreme diets. Lipid intake was also more tightly regulated in the choice experiment, as its variation across the three food-pairing treatments was narrower than that of protein intake (Figure 3B). Our current understanding of how honeybees perceive and regulate their protein (amino acid) and lipid (fatty acid) intake is poorly understood, especially compared to carbohydrates.^18^^,^^19^^,^^49^ However, there are similarities between how all animals regulate and process nutrients.^1^^,^^2^^,^^6^^,^^7^ For example, in humans, the presence of fat in the small intestine slows gastric emptying, stimulates the release of various gastrointestinal hormones, and suppresses appetite and energy intake.^61^ These effects are largely due to the digestion of fats (triacylglycerides) into fatty acids61. Additionally, high fat diets can significantly alter gut microbiota.^62^

It is also worth noting that our study was conducted using nurse bees without brood. It is possible that the presence of brood may shift the nurse bees’ protein-lipid ratio preferences, as has been observed for ants.^63^ However, the P30:L20 diet produced the largest HPG, so if the best predictor of larval performance is HPG size, we suspect that the P:L intake target for nurse bees would not change even in the presence of brood. Alternatively, food quality (e.g., amino acid or fatty acid profiles) may matter more than quantity. Currently we do not know how diet quality influences the composition of brood food and royal jelly.^20^ Experiments feeding nurse bees diets that differ in their protein-lipid content, in the presence and absence of brood, would establish whether the chemical composition of royal jelly is relatively standard (as suggested by Wright et al.^18^), or if it varies as a function of environmental inputs (pollen quality) and/or stimuli (brood).

Honeybees are indispensable for the cross-pollination of over 100 different fruit, vegetable, seed, and nut crops.^64^^,^^65^ Despite their economic importance, the number of colonies has dropped dramatically in the last decade, threatening the production of bee-dependent crops. Respondents of a recent national colony loss survey reported their winter losses to be 30–50%, with over 50% of the 3,000+ surveyed operations attributing their losses in part to poor nutrition.^22^ These statistics highlight the urgent need to improve honeybee nutrition and implement better apicultural and agricultural practices that improve colony health and productivity. Until recently, researchers thought that pollen was collected by bees for its protein content alone,^27^^,^^66^^,^^67^^,^^68^^,^^69^ but our results suggest that for nurse bees, pollen lipid content is also important. In particular, the amount and type of fatty acids contained in pollen are relevant for bee health, including task transitioning and performance.^20^^,^^69^^,^^70^ Given the significant variation in the types of pollen available in agricultural, rural (natural, not used for crop production), and urban settings,^27^ and because each pollen type has a unique nutritional profile, the multidimensional nutritional space available to honeybees is likely to vary widely between habitats. Thus, the fitness implications for managed and free-living bees consuming these resources are expected to diverge substantially among landscapes. For all the reasons stated above, landscapes should be managed in a way that provides pollen resources that are best aligned with pollinator health.^71^^,^^72^ Interestingly, the P:L ratios of pollen collected by bees in the field varies as a function of life-history,^45^^,^^46^ and they appear to have an underlying phylogenetic signature.^46^ This type of information provides important nutritional insights that can potentially be used to manage habitats and/or locations based on the existing palynivore diversity and abundance. Finally, our results may have important implications for predators and omnivores, which also eat foods that contain large amounts and varying ratios of dietary protein and lipids. Perhaps the strict feeding rule practiced by honeybees is the norm for animals that eat foods rich in protein and lipids? Compared to protein-carbohydrate NGF studies, relatively few protein-lipid NGF studies exist for any animal. Therefore, more studies on palynivore, predator, and omnivore foraging behavior will reveal the extent to which globally regulatory rules exist for protein-lipid regulation, as they do for protein-carbohydrate regulation.^4^^,^^5^^,^^73^^,^^74^

### Limitations of the study

Our results revealed new insights regarding nurse honeybee protein and lipid regulation, but we acknowledge three limitations of our study. First, as covered in the Discussion, our range of treatments did not include diets with highly biased protein content. Second, we did not manipulate protein or lipid quality. Pollen is a highly complex, multidimensional resource,^18^^,^^19^ whose species-specific qualitative makeup —often following phylogenetic patterns^46^^,^^47^^,^^75^— can profoundly influence honeybee nutritional physiology. Third, pollen contains multiple lipid classes, including triacylglycerols, free fatty acids, sphingolipids, galactolipids, glycerophospholipids, and sterols.^54^ Because triacylglycerols are the most abundant lipid class, they were our primary focus. Notably, the diets used in our study did not contain sterols, which are crucial for insect growth and development,^76^^,^^77^ including that of bees.^78^^,^^79^ The diets also lacked pollen phytochemicals, which can stimulate immune responses to pathogen infections and pesticide exposure.^80^^,^^81^ Future studies that systematically vary the concentrations and ratios of key nutrients and bioactive compounds – and explore their interactive effects on foraging preferences, nutrition, and colony health – will be essential for achieving a truly holistic understanding of honeybee nutrition.

## Resource availability

### Lead contact

Further information and requests for resources should be directed to and will be fulfilled by the lead contacts, Professors Juliana Rangel (jrangel@tamu.edu) and Spencer T. Behmer (s-behmer@tamu.edu).

### Materials availability

This study did not generate new unique reagents.

### Data and code availability

Any additional information required to reanalyze the data reported in this paper is available from the lead contact upon request. This paper does not report original code. Any additional information required to reanalyze the data reported in this paper is available from the lead contact upon request, subject to their approval.

## Acknowledgments

We would like to thank Omar Khan and Mary Beth Buchman for their help in the field and with cage maintenance. This work was supported in part by a USDA-NIFA Predoctoral Fellowship (award no. 2019-07304) to P. Lau, as well as research grants to P. Lau, A.N.P., and J.R. from the North American Pollinator Protection Campaign, the Eastern Apicultural Society of North America, and the Louisiana Beekeepers Association. The USDA is an equal opportunity employer.

## Author contributions

P. Lau, P. Lesne, A.N.P., S.T.B., and J.R. designed the research; P. Lau, P. Lesne, A.P., C.G., and J.G. performed the research; P. Lau, P. Lesne, A.N.P., S.T.B., and J.R. analyzed the data; P. Lau, P. Lesne, A.N.P., S.T.B., and J.R. wrote the paper.

## Declaration of interests

The authors declare no competing interests.

## STAR★Methods

### Key resources table

**Table:** 

REAGENT or RESOURCE	SOURCE	IDENTIFIER
**Biological samples**		

Honeybee (*Apis mellifera*) workers	Janice and John G. Thomas HoneyBee Facility (Texas A&M University, Bryan, TX, USA)	NCBI:txid7469

**Experimental models: Organisms/strains**		

Honeybees (*Apis mellifera*)	Janice and John G. Thomas HoneyBee Facility (Texas A&M University, Bryan, TX, USA)	N/A

**Software and algorithms**		

R Software (3.2-3)	RCoreTeam^82^^,^^83^	www.r-project.org
Excel (2025)	Microsoft, Redmond, USA	www.microsoft.com

**Other**		

Queen rearing plastic cups	Jz’s-Bz’s Honey Co., Santa Cruz, CA, USA	https://jzsbzs.com
Isolated soy protein powder	Nuts.com	https://nuts.com
Vanderzant vitamin mix for insects	MP biomedicals	https://www.mpbio.com/us/
Organic flax cooking oil (Linseed oil)	Carrington farms	https://carringtonfarms.com/
Cellulose	Millipore Sigma	https://www.sigmaaldrich.com/US/en

### Experimental model and study participant details

The honeybee (*Apis mellifera*) colonies used in this study were maintained at the Janice and John G. Thomas HoneyBee Facility (Texas A&M University, Bryan, TX, USA) following standard beekeeping techniques.

### Method details

Two separate experiments were performed: (1) a no-choice experiment with one diet provided per experimental cage, and (2) a choice experiment with two nutritionally complementary diets provided per experimental cage. Both experiments followed a common general protocol (described in the section below, titled “nurse bees, experimental arenas, and general protocol”) that required the use of artificial diets (described in the section below, titled “artificial diets”). The specifics of the no-choice and choice experiments are described in the sections below, titled “no-choice NGF experiment” and “choice NGF experiment,” respectively.

#### Nurse bees, experimental arenas, and general protocol

For both no-choice and choice NGF experiments, nurse bees were obtained by randomly selecting frames of capped brood from six source colonies located at the Janice and John G. Thomas HoneyBee Facility (Texas A&M University, Bryan, TX, USA) during the summer of 2019. The frames were incubated at 34°C for 24 h. Next, cohorts of newly emerged bees were collected, pooled, and randomly distributed in clear plastic cages (11.43 cm diameter x 13.97 cm height); each cage had *ad libitum* supplies of water and sucrose solution (50% w/v) provided by gravity feeders. The food dishes, which were two queen-rearing plastic cups (Jz’s-Bz’s Honey Co., Santa Cruz, CA, USA), were filled with artificial diet (details below). These food dishes were left to equilibrate to room humidity levels for 24 h and then weighed to the nearest 0.1 mg. They were then placed into the experimental cages for 24 h. After this 24 h period, all the food dishes were removed and replaced with a new food dish. Each original food dish was allowed to equilibrate to room humidity levels for 24 h before being reweighed. This procedure followed the methods of Behmer et al.^84^ and allowed us to measure the total amount of food consumed, plus protein and lipid intake, by the bees in each cage. The death of any bee was recorded, and all dead individuals were removed from the cage daily. We repeated this procedure for six consecutive days. This time frame corresponds to the period during which nurse bees develop their hypopharyngeal glands and feed the developing larvae with brood food.^51^ At the end of day six, the remaining live bees in each cage were collected in a 50 mL conical tube and stored at −20°C for subsequent analysis. All experiments were conducted in an incubator kept at 34°C and 60% relative humidity.

#### Artificial diets

We made two major changes to the methods of the NGF protein-lipid studies previously described in Stabler et al.^33^ and Vaudo et al.^41^ First, we presented nurse honeybees with solid diets designed to resemble pollen. Second, linseed oil (a triacylglyceride) was chosen as the lipid source because: 1) it has a high proportion of relevant fatty acids and its high omega-3-to-omega-6 fatty acid ratio,^85^^,^^86^ and 2) triacylglycerides are the dominant lipid form in pollen lipid droplets.^54^ In total we created five artificial diets (similar to those used in Powell et al.^87^) that spanned a range of protein (P) and lipid (L) amounts reported in honeybee collected pollen^18^^,^^45^^,^^46^: (1) P35:L15; (2) P30:L20; (3) P25:L25; (4) P20:L30; and (5) P15:L35. Isolated soy protein powder was chosen as the protein source because of its amino acid profile best matches the amino acid needs of honeybees.^85^ The protein plus lipid content of each diet was 50% of the total biomass (wet weight). The remaining 50% of each diet was a carbohydrate-vitamin-filler blend that was kept constant across the diets. The bulk of this (39.5% wet mass) was a 50% sucrose solution; sucrose was chosen as the carbohydrate source because it is the most abundant sugar in nectar.^88^ Vanderzant vitamin mix (0.5%) was the vitamin source. Cellulose (10%) was chosen as a filler because it provided the appropriate texture to the food and is a natural constituent of pollen.^89^ The water-soluble vitamin mix was dissolved in the sucrose solution prior to combining it with the protein powder, linseed oil, and cellulose. As an example, 100 g of the P30:L20 diet contained: 1) 30 g of protein, 2) 20 g of lipid, 3) 39.5 mL of a 50% sucrose solution, 4) 0.5 g Vanderzant vitamin mix, and 5) 10 g of cellulose. The diets were mixed thoroughly using a KitchenAid tilt-head stand mixer. One final note, our five diets were not isocaloric; as lipid content decreased, caloric density also slightly decreased (see Document S1).

#### No-choice NGF experiment

In this no-choice experiment, each test cage had two queen-rearing plastic cups containing the same artificial diet (i.e., either P35:L15, P30:L20, P25:L25, P20:L30, or P15:L35) in sufficient amounts for all bees to feed from the cups, *ad libitum*. The amount eaten from each food dish was recorded daily for six days (see above for details). There was also a no-food, negative control treatment; in these cages, nurse bees were provided access to water and a 50% sucrose solution (50% w/v), but they received no artificial diet. For each treatment, there were twelve replicate experimental cages, each containing 30 nurse bees. All treatments and replicates were run as a single trial.

At the end of the experiment, we euthanized all remaining 10-day-old bees so that we could measure HPG acinus size and total lipid content across the different treatments.^52^ HPG acinus size is used as a proxy to assess the nutritional status of nurse bees,^51^^,^^90^ while total lipid content can be used as a correlate of a bee’s physiological condition.^91^ To measure HPG size, a single bee was randomly selected from each experimental cage (n = 12 per treatment, for a total of 72 bees) and her HPGs were dissected to measure the diameter of 10 acini per bee under a dissecting microscope.^86^ To measure total lipid content, we randomly selected another bee (n = 12, 72 bees total) and her lipids were extracted and quantified using the Folch method.^92^

#### Choice NGF experiment

In this choice experiment there were three food-pairing treatments (FPT) varying in protein-to-lipid content (FPT 1: P20:L30 with P30:L20; FPT 2: P20:L30 with P35:L15; and FPT 3: P25:L25 with P35:L15). Each experimental cage had two queen-rearing plastic cups, each containing one of the two diets in the pairing. Each food-pairing treatment contained nutritionally complementary diets. We excluded the P15:L35 diet in choice experiments because we felt it was too lipid biased. The amount eaten from each dish was recorded daily for six days, as described above. Each diet was offered in sufficient amounts such that all bees could feed from each cup *ad libitum* without running out of food. For each treatment there were twelve replicate experimental cages, each containing 25 nurse bees. All treatments and replicates were run as a single trial. As in the no-choice experiment, we recorded daily mortality and final survival for each experimental cage. We also removed all dead bees daily, collected all bees that were alive at the end of the trial, and stored them at −20°C in 50-mL tubes for subsequent analysis.

### Quantification and statistical analysis

#### Total food and macronutrient consumption

For all trials, total daily food consumption was measured for each dish by taking the difference between the initial and the final dry mass of individual food dishes. Because we knew the proportion of protein and lipid in each diet, and the total amount of food consumed from each food dish, we could calculate protein and lipid intake from each food dish. Daily consumption was calculated on a per capita basis by dividing food, protein, or lipid intake, by the number of nurse bees that were still alive in the experimental cage on that day.

For the no-choice experiment, the total per capita consumption was compared across diet treatments using one-way ANOVAs followed by pairwise Tukey HSD tests. We also used ANOVAs to compare the combined collected amounts of protein and lipid (the two dependent variables), summed over the duration of the experiment, across the three different food-pairing treatments; differences in protein and lipid intake across the five treatments were assessed using pairwise Tukey HSD tests.

For the choice experiment, the total per capita consumption was compared between the three food-pairing treatments using a mixed ANOVA (R package *rstatix*^93^). We included a between-subject independent variable in the model to reflect that there were two diets in each experimental cage (e.g., one relatively high in protein, the other relatively high in lipid), and treated the experimental cage as a random variable. This analysis was followed by pairwise Bonferroni corrected *t*-tests, for a total of three food-pairing treatment comparisons. In contrast to the no-choice data, the combined collected amounts of protein and lipid per cage were compared across treatment groups using a MANOVA; univariate ANOVA tests of protein and lipid intake were followed by pairwise Tukey HSD tests. We compared the self-selected P:L ratios for the three food-pairing treatments using a one-way ANOVA, followed by Tukey HSD tests. The P:L ratios were calculated for the entire experiment by dividing the total protein intake by total lipid intake.

We further explored the consumption data from the choice experiment to assess to what extent bees were actively regulating their dietary protein-lipid intake. First, we tested whether bees showed a preference for one food dish over the other. For every experimental cage, we calculated the difference in the total amount of food collected from each of the two dishes. This difference was then compared against a null value of zero (indicative of no preference) using one-sample *t*-tests; *p*-values were adjusted using a Bonferroni correction (for a total of three comparisons, one for each food-pairing treatment). Second, we tested whether bees were actively regulating their P:L intake using a compensatory mechanism (i.e., correcting the P:L imbalance of one food by eating the other complementary food in an appropriate amount), as opposed to eating the two available foods based solely on their individual composition (i.e., as would happen in a no-choice context), as done by Hendriksma and Shafir.^32^ To do this, we created a theoretical choice experiment dataset using the daily per capita amounts of food collected from the no-choice experiment. This new theoretical dataset reflected the amount of food that would have been collected in a choice experiment if collection was independent from the influence of the other food. Theoretical consumption values were estimated by dividing the average per-capita diet intake from the no-choice experiment by two, reflecting the number of food dishes presented in the choice experiment. This comparison allowed us to assess whether actual consumption in the choice experiment deviated from the theoretical expectation, revealing potential nutrient intake optimization strategies. We conducted two comparisons. First, we used *t*-tests with Bonferroni adjusted *p*-values to compare the theoretical and experimental food consumption across the three food-pairing treatments. Second, we applied the same statistical approach to compare the theoretical and experimental P:L ratios for each food-pairing treatment.

#### Survival, HPG size, and total body lipid

Bee survival under the no-choice and choice experiments was compared using cox mixed effect models (R package *coxme*^82^), with the diet treatment as the fixed variable and the experimental cage as a random effect. The average diameter of twelve HPG acini was calculated per bee, and these averages were compared between diet treatments using ANOVAs followed by pairwise Tukey HSD tests. The HPG acinus sizes were log transformed to comply with the assumptions of parametric statistics. The relationships between average HPG acinus size and protein and lipid intake were assessed using linear regressions. Individual lipid content was than compared across treatments using ANOVAs, followed by pairwise Tukey HSD tests for post hoc analysis.
