# Supplementary material for: Do not compromise: Nurse honeybees practice strict protein-lipid regulation
Source: iScience. 2025 Jun 17;28(7):112895. doi: 10.1016/j.isci.2025.112895 (PMC12266554; doi:10.1016/j.isci.2025.112895)
Supplement: Data S1. Figures S1–S3 and Document S1 [file mmc1.pdf]

iScience, Volume *28*

Supplemental information

Do not compromise: Nurse honeybees practice strict protein-lipid regulation

Pierre Lau, Pierre Lesne, Alexandria N. Payne, Cora Garcia, Jordan Gomez, Spencer T. Behmer, and Juliana Rangel

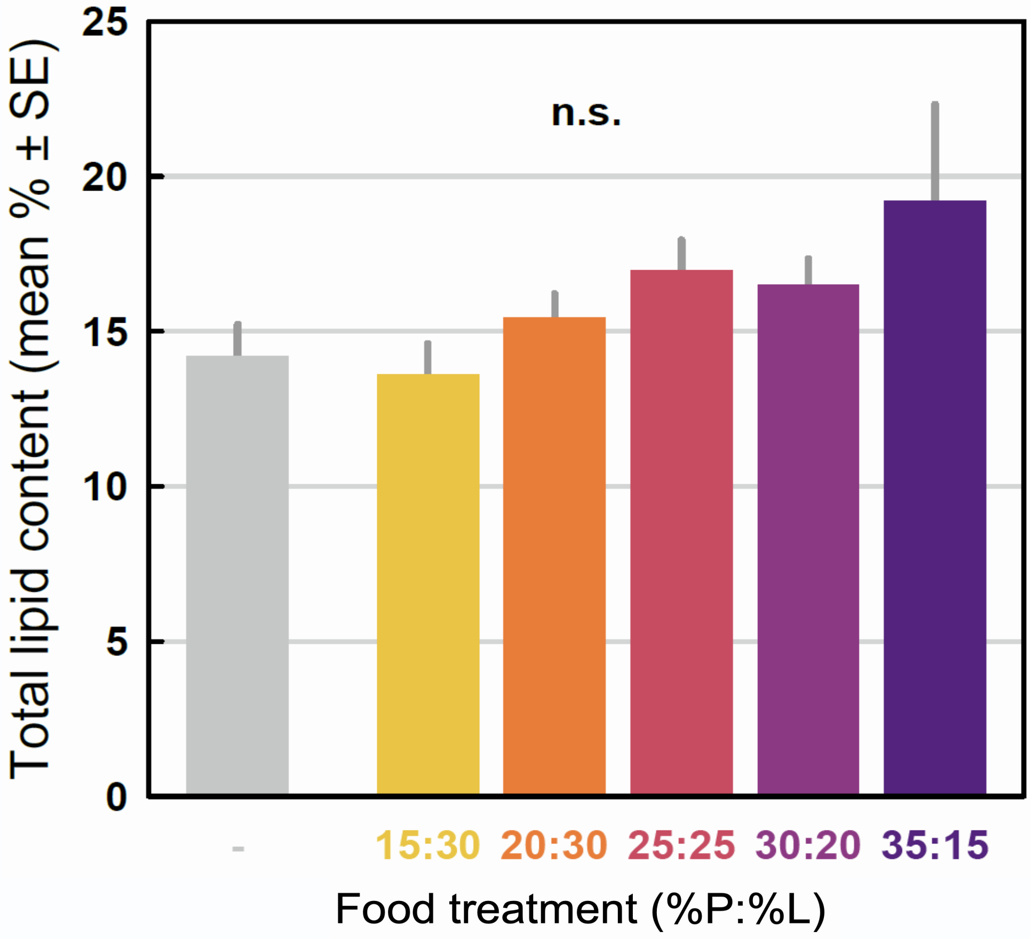

Figure S1. Total lipid content (mean ± SEM) per nurse bee after being fed diets with different protein-lipid content during the no-choice experiment. There were no significant differences in the total lipid content (mean ± SEM) extracted from nurse bees given diets that varied in their protein-lipid (P:L) ratio. Diets represented in yellow are more lipid rich; diets represented in purple are more protein rich. The gray bar (-) represents the negative control treatment group; nurse bees were only fed sucrose syrup.

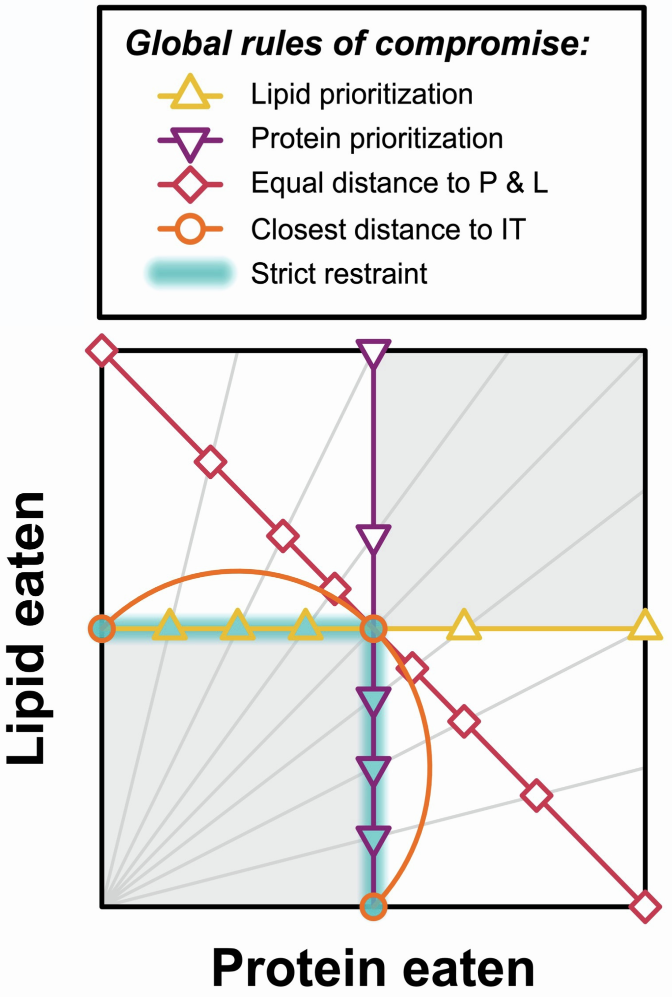

Figure S2. Global feeding rules in the Nutritional Geometry Framework. Graphical representation of the theoretical relative amounts of protein and lipid eaten by an organism following different rules under a no-choice experimental context (modified from Simpson et al. [6]). The grey lines radiating from the origin represent the Protein-Lipid ratio (P:L) of different diets; the point centered in the middle is a theoretical "intake target" (IT; the optimal blend of protein and lipid). Five outcomes are represented: (1) Eat until the protein requirement is met, irrespective of lipid intake (purple triangles, purple line); (2) eat until the lipid requirement is met, irrespective of protein intake (yellow triangles, yellow line); (3) eat until the sum of protein and lipid consumed equals the sum at the intake target ("equal distance" rule); (4) eat until the geometrically closest distance to the intake target is reached on the food rail ("closest distance" rule); and finally, (5) eat until the intake target level of either protein or lipid is reached (blue highlight). We call this the "strict restraint" rule.

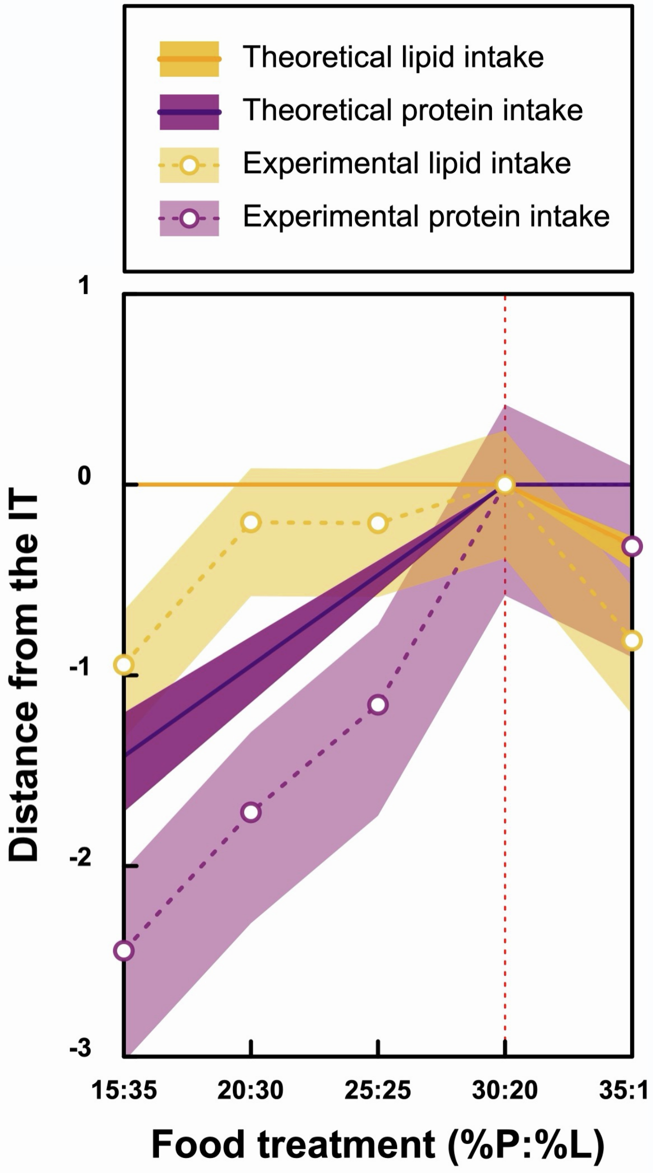

Figure S3. Graph comparing the theoretical and experimental distance from the protein-lipid "Intake Target" (IT) under the "strict restraint" rule. The IT (represented by the thin vertical dashed red line) was inferred from the no-choice and choice experiments. The theoretical data are shown as dark solid lines (purple = protein, orange = lipid); the experimental data are shown as dashed lines. The error ribbon around the theoretical values represents the maximum variation associated with the error around the intake target. The error ribbon around the experimental data represents the standard error of the average distance of the eaten diet to the average IT. This visualization suggests that honey bee nurses regulate lipid intake more tightly than protein intake.

Document S1. Our treamtents relative to reported P:L ratios from pollen (from Vaudo et al.[45]).

P = lipid content of artifical diets (%); L = lipid content of artifiical diets (%); PL value = the calculated P:L ratio for a given diet.
Orange represents our actual diets; Green represents gradual variation away from our diets (these diets are shown for reference only, and to show distribution of P:L ratios over a continuum)
Unadjt # = the count of observed pollen P:L ratios from Vaudo et al.[45]).
Adj # = the count of observed pollen P:L ratios from Vaudo et al.[45] after correcting for an underestimation of lipid content (we adjusted lipid values upward 20%).

Treatments	P (%)	L (%)	PL value	Unadjt #	Adjt #	% Unadjt	% Adjt
	2.5	47.5	0.05				
lipid-biased	5	45	0.11	1	1	1.2%	1.2%
	7.5	42.5	0.18				
lipid-biased	10	40	0.25	1	1	1.2%	1.2%
	12.5	37.5	0.33				
our treatment	15	35	0.43	3	4	3.7%	4.9%
	17.5	32.5	0.54				
our treatment	20	30	0.67	13	16	15.9%	19.5%
	22.5	27.5	0.82				
our treatment	25	25	1.00	12	13	14.6%	15.9%
	27.5	22.5	1.22				
our treatment	30	20	1.50	15	16	18.3%	19.5%
	32.5	17.5	1.86				
our treatment	35	15	2.33	12	12	14.6%	14.6%
	37.5	12.5	3.00				
protein-biased	40	10	4.00	19	16	23.2%	19.5%
	42.5	7.5	5.67				
protein-biased	45	5	9.00	6	3	7.3%	3.7%
	47.5	2.5	19.00				
			total	82	82		

Caloric value of our five experimental diets.			
Treatment	P (%)	P (%)	calories
P15:L35	15	35	375
P20:L30	20	30	350
P25:L25	25	25	325
P30:L20	30	20	300
P35:L15	35	15	275

Our coverage	67.1%	74.4%
N-rich	30.5%	23.2%
L-rich	2.4%	2.4%
